# Evaluation of analytical methods for the determination of the physicochemical properties of fermented, granulated, and roasted cassava pulp ‐ gari

**DOI:** 10.1002/fsn3.363

**Published:** 2016-04-05

**Authors:** Cornelia Felber, Yaovi Ouézou Azouma, Marcus Reppich

**Affiliations:** ^1^ Faculty of Mechanical and Process EngineeringAugsburg University of Applied SciencesAn der Hochschule 186161AugsburgGermany; ^2^ Agricultural Engineering DepartmentUniversity of LoméBP 1515LoméTogo

**Keywords:** Analytical methods, cassava, food quality, food safety, Gari, physicochemical properties

## Abstract

Simple but reliable methods for the determination of the physicochemical properties of gari were evaluated for the parameters, such as grain size, bulk density, swelling index, moisture content, gross calorific value, cyanide content, and acidity content. The grain sizes were between 525 and 928 *μ*m (weighted means), the bulk densities between 0.541 and 0.699 g/cm³, and the swelling indices between 3.21 and 4.33. The moisture contents ranged from 4.30 to 9.19%. The gross calorific values were found between 15.45 and 15.82 kJ/g. The cyanide contents were between 0 and 4.8 ppm. The acidity contents varied among 0.55 and 1.62%. Correlation tests verified the influences of the grain size and the moisture content on the acidity content with a probability of 99.9%. The methods were regarded as suitable and adaptable for the application in small and medium cassava‐processing industries with special regard to the respect of the consumer`s health safety.

## Introduction

Cassava (*Manihot esculenta crantz*) is a major staple food in Africa, Asia, and Latin America and accounts for many people in the developing countries to about 20% of their total energy intake (Hillocks et al. 2002). The plant consists of 6% leaves, 44% stem, and 50% roots. Hereof particularly the root is consumed as food. It contains 60–66% moisture and 32–35% carbon hydrates. The plant's protein content is with 0.4–0.6% very low as well as its fat content of 0.1–0.3%. Especially its high moisture content, as well as recognizable amounts of linamarin result in the necessity to process cassava immediately after harvest into different storable products (Heuberger 2005). Thus, in Africa 75% of all cassava is processed into gari, which is won by pressing (and sometimes fermenting), granulating, and roasting the peeled and grated cassava roots (smallstarter.com, 2013). The processing methods of gari vary among different producers. Hence, not only the final product gari is dependent on the used cassava variety, the maturing process of the plant including climate and soil conditions, and the harvesting time but also the different production steps. The Agricultural Engineering Department of the University of Lomé is working on the promotion of Small and Medium Agrofood Industries (SMAI) in Togo. Thus, in order to optimize the production processes and to improve the qualifying properties, simple and cheap methods have to be defined to compare different products according to certain parameters influencing the quality of the final product. The chosen quality parameters are the grain size distribution, the bulk density, the swelling index, the moisture content, the gross calorific value, the cyanide content, and the acidity content expressed as lactic acid. Several methods were regarded according to their adaptability for gari and the operability in Africa. Hence, this essay describes simple and cheap methods to be used for a determination of the named properties within small and medium cassava‐processing industries in order to improve the quality of gari and respect the health safety of the consumers of gari. Moreover, correlations between the parameters can influence the results. Hence, the interaction between the factors grain size and moisture content and their impact on the acidity content are exemplarily observed within this examination. Table 1 depicts the used nomenclature.

**Table 1:** Used nomenclature

Symbol	Meaning	Unit
BD	bulk density	g/cm³
*c*_W_	heat capacity of water	J/gK
*d*	diameter	*μ*m
dwt	dry weight	g, kg
fwt	fresh weight	g, kg
*h*	humidity, moisture content	%
*H*_h,B_	gross calorific value of benzoic acid	J/g
*H*_h,F_	gross calorific value of light fuel oil	J/g
*H*_h,G_	gross calorific value of gari	J/g
*H*_h,I_	gross calorific value of the ignition wire	J/g
*i*	running index	−
*K*	calorimeter constant	J/K
*m*	mass	g
*m*_B_	mass of the benzoic acid pellet	g
*m*_F_	mass of light fuel oil	g
*m*_G_	mass of the gari sample	g
*m*_I_	mass of the ignition wire	g
*m*_M_	mass of the gari‐fuel mixture	g
*m*_W_	mass of the water in the water bain	g
*n*	number of trials	−
*P*	Probability	%
SI	swelling index	−, %
*V*	volume	mL, cm³
$\overset{¯}{x}$	arithmetic average	(different units)
${\overset{¯}{x}}_{3_{i}}$	weighted average of the grain size	*μ*m
*ΔT*	temperature difference	K

## Materials and Methods

### Materials

#### Gari samples from the south of Togo

**Figure 1:**
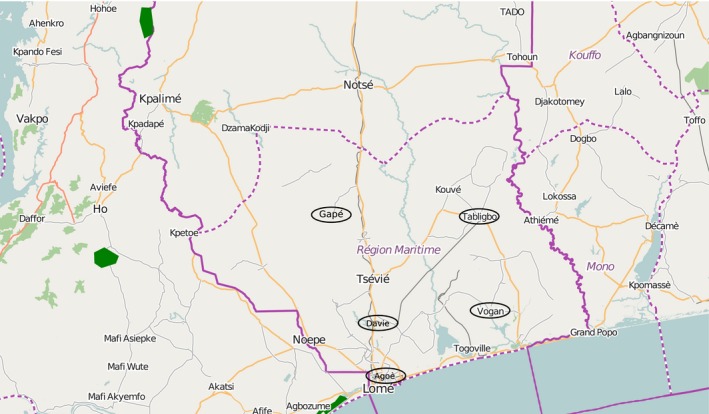
Sample collection in Togo Maritime [adapted from (OpenStreetMap, 2014)].

The following evaluation of possible methods bases on 14 samples collected in the south of Togo. Therefore, the investigation units include two gari processing centers at Davie and Gapé as well as three local markets at Agoè in Lomé, Tabligbo, and Vogan to cover the most important markets of gari in Togo. The samples derive from April and May 2014 and are named according to their place of origin and order of collection: Agoè I, Agoè II, Agoè III, Agoè IV, Davie I, Davie II, Gapé‐Batekpo, Gapé‐Nyassivé, Tabligbo I, Tabligbo II, Tabligbo III, Vogan I, Vogan II, and Vogan IV. Figure 1 depicts the local origin of the samples marked with circles.

#### Used laboratory equipment

For the analysis of the chosen parameters, the following laboratory equipment was necessary (see Table 2).

**Table 2:** Laboratory equipment used for the described measurements including all specifications

Equipment	Specifications
Sieve stack (Retsch, AS 200 basic)	Measuring range: 20 *μ*m–25 mmAmplitude: 0–3 mmTime slot: 1–60 min
9 sieve inlets (Retsch, AS 200 basic)	Sizes: 125 *μ*m, 180 *μ*m, 250 *μ*m, 355 *μ*m, 500 *μ*m, 710 *μ*m, 1000 *μ*m, 1400 *μ*m, 2000 *μ*mDiameter: 200 mm
Technical balance (Mettler Toledo, PM 4600 DeltaRange)	Measuring range: 0.5–4100 gAccuracy: ±0.01 g
Analytical balance (Sartorius)	Measuring range: 0.00005–210 gAccuracy: ±0.01 mg
Hot‐air cabinet (Heraeus, UT 6060)	Nominal temperature: 300°CPower consumption: 1.50 kW
Spectrophotometer (Hach Lange GmbH, CADAS 200)	Extinction: − 3E to+ 3ETransmission: 0–100%Wavelength: 190–1100 nm
Static‐jacket calorimeter (Phywe Systeme GmbH & Co.KG); temperature measurement included	Device modified for university usageAccuracy of the temperature measurement: ±0.20 K
Titration device (Hirschmann, solarus 50)	Draw‐up volume: 50 mLAccuracy: ±0.01 mL

Moreover, several sizes of glass vessels, pipettes, and automatic micropipettes, as well as pestle and mortar were in use.

### Methods

The chosen methods are the best according to the required performance skills, necessity of laboratory equipment, the availability of material, the needed expenses, accuracy, and robustness. The results are presented as arithmetic averages out of every measurement. All trials followed a randomized sequence.

#### Grain size distribution, bulk density, and swelling index

**Figure 2:**
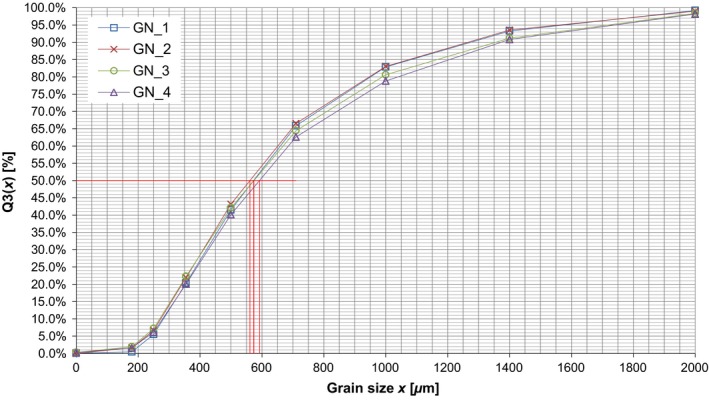
Frequency distribution of the sample Gapé‐Nyassivé for four trials.

The grain size distribution was examined by a dry sieving method using a sieve stack according to ISO 3310 (Deutsches Institut für Normung, 2001). The used sieves had mesh sizes between 125 *μ*m and 2000 *μ*m. The sieving time was 10 min and 15 min, respectively. Each sample was determined four times. For the comparison serve the medians and the weighted means. The median defines the intersection point of the cumulative distribution curve with the 50% horizontal. Figure 2 shows the distribution for all four trials exemplarily for one sample and where to measure the median. In this case the four median values can be found at 573 *μ*m, 561 *μ*m, 574 *μ*m, and 592 *μ*m, which result in an average of 575 *μ*m.

The weighted mean can be calculated according to the following equation (1) (Stiess 2009):

(1) $${\overset{¯}{x}}_{3}=\sum_{i=n}^{n}d_{m,i}\cdot \mu_{3,i}=\sum_{i=1}^{n}\frac{\left(d_{i-1}+d_{i}\right)}{2}\cdot \mu_{3,i}$$

The International Association of Official Analytical Chemists recommends measuring the bulk density by using a mass of 10.00 g gari being put into a 50‐mL measuring cylinder (AOAC, 1990). Before metering the volume of the sample, the cylinder was being tapped 40 times. The bulk density equals the ratio of the sample mass to its volume:

(2) $$\text{BD}=\frac{m}{V}$$

The method of Ukpabi and Ndimele of 1990 suggests determining the swelling index by putting a mass of 10.00 g gari into a measuring cylinder and adding a volume of 50.0 mL of distilled water (Owuamanam et al. 2011). After a rest of 4 h, the swelling index corresponds to the ratio of the swollen volume to the initial volume (see equation (3)):

(3) $$\text{SI}=\frac{\text{swollen volume}}{\text{initial volume}}$$

#### Moisture content

The moisture content was quantified by a thermogravimetric method using a hot‐air cabinet (Wernecke 2008). A mass of 10.00 g of the unprepared sample was weighed into a glass jar and placed into the oven at 105°C for 48 h. After 24 h, an intermediate weighing step is necessary for the control. If the weight has still changed after 48 h, the drying time should be increased. Each sample was metered five times. The moisture content and drying loss, respectively, equals the ratio of the dry weight to the fresh weight of the sample:

(4) $$h=\frac{\text{dwt}}{\text{fwt}}$$

#### Gross calorific value

The gross calorific value was measured using a static‐jacket calorimeter according to DIN EN 15400 (Deutsches Institut für Normung, 2011). Therefore, about 320 mg of the thoroughly grinded sample was weighed into the combustion cup. Additionally, some drops of light fuel oil (around 80 mg) with well‐known gross calorific value were added to improve the combustion process. The exact masses are necessary for the calculation and their possible sizes depend on the used calorimeter. A special fixture holds the combustion cup and enables the ignition wire to connect the sample with the two electrodes. Then the whole construction is set into the jar, closed tightly, and filled up with oxygen up to a pressure of 10 bars. After connecting the two electrodes, the calorimetric bomb slides into a water bain, which contains 1000 g of distilled water. A temperature sensor takes the temperature of the water and its increase during 15 min after the ignition of the bomb. Five different samples were metered each four times. An additional performance with a benzoic acid pellet is necessary to compute the calorimeter constant.

**Figure 3:**
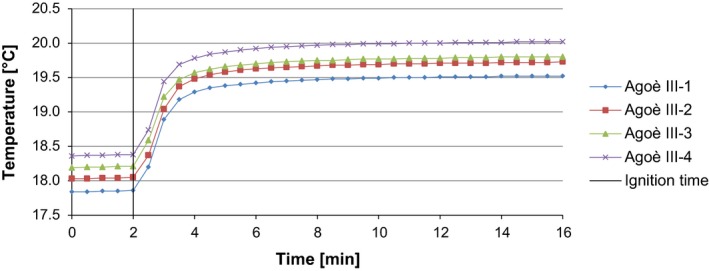
Temperature profile of the determination of the calorific value of Agoè III.

If the combustion takes place as expected, the results of the gross calorific value are first of all found in the temperature profile. This profile includes a previous period, the extreme temperature shift, and the backlash. The temperature profile is shown exemplarily for one sample in Figure 3.

As already mentioned, the performance of the experiment with a benzoic acid pellet instead of the sample lead to the calculation of the calorimeter constant. Therefore, the gross calorific value of the benzoic acid pellet and its mass, as well as the gross calorific value of the ignition wire and its weight are needed. Moreover, the measured temperature increase, the mass of the water, and its specific heat capacity are essential for the calculation (see equation (5)):

(5) $$K=\frac{H_{h,B}\cdot m_{B}+H_{h,I}\cdot m_{I}}{\Delta T}-m_{w}\cdot c_{W}$$

The gross calorific value of the mixture (sample and fuel oil) can be computed as shown in equation (6):

(6) $$H_{h,M}=\frac{\left(m_{W}\cdot c_{W}+K\right)\cdot \Delta T-H_{h,I}\cdot m_{I}}{m_{M}}$$

Finally, the gross calorific value of the sample can be calculated by subtracting the part of the fuel oil as depicted in equation (7):

(7) $$H_{h,G}=\frac{H_{h,M}\cdot m_{M}-H_{h,F}\cdot m_{F}}{m_{G}}$$

#### Cyanide content

The cyanide content was determined using the picrate paper kit developed by Egan, Yeoh, and Bradbury in 1998 (Egan et al. 1998; Bradbury 2009). The kit contains a portable plastic balance for weighing 100 mg, two graduated 1‐mL plastic pipettes, 30 clear plastic vials with screw lids, 100 pH 6 buffer and linamarase papers, 100 picrate papers on clear plastic strips, one color chart with 10 shades of color, which correspond to 0–800 ppm total cyanide, and 10 pink standard papers containing linamarin equal to 50 ppm cyanide. For the measurement, the vessel was filled with a buffer and enzyme paper, 100 mg of the thoroughly grinded sample, and 5 mL of distilled water, as well as a yellow picrate paper, and was closed tightly. Then the vial has to rest for 20 h at room temperature. The color of the picrate strips was compared to the color chart. By laying the strip into 5.0 mL of water and leaving it for 30 min at room temperature the photometric determination of the cyanide content becomes possible. The total cyanide content in ppm corresponds to the absorbance of the solution at 510 nm multiplied by 396 (Bradbury et al. 1999).

#### Acidity content

The acidity content expressed as lactic acid equivalents is measured by a titration method using distilled water as solvent, sodium hydroxide as leach, and phenolphthalein as indicator (Pearson 1973; Lees 1975 and AOAC 1990). A mass of 5.00 g of the grinded sample was solved in 100.0 mL of distilled water and agitated for 1 h. Before stirring again, the sample rested for 24 h. After filtration, a volume of 20.0 mL of the filtrate was titrated with 0.05 molar sodium hydroxide and a few drops of phenolphthalein as indicator. The titration is completed when the color of the solution turns pale rose; the color change has to be stable for at least 30 sec. The total titrable acidity expressed as percentage lactic acid can be computed as shown in equation (8) out of the titration volume. The formula already includes the moisture correction:

(8) Corrected TTA%lactic acid=0.450·titration volume [mL]/(1−h)

#### Correlations between the factors grain size, moisture content, and acidity content

Possible correlations among the factors grain size, moisture content, and acidity content were examined using the Design of Experiments method (Cobb 1998). The acidity content was determined under two treatments with each two levels: for the factor grain size, the two levels are fine and coarse grains, whereas the factor moisture content varied between dry and humid. The chosen units for the experiment performance derive from a sample that had been divided into two mass fractions (Vogan I and Vogan II). With the intention of defining the moisture content, the samples have to be dried as described above. A two‐way basic factorial design with four replications is the model for the correlation tests. All measurements take place in randomized sequence.

## Results and discussion

Table 3 shows the results of the 14 samples for all parameters. All results are presented as arithmetic averages out of all trials.

**Table 3:** Computed results of the measurements for 14 samples and all 7 parameters presented as arithmetic averages out of the performed trials

Sample	Weighted mean [*μ*m]	Bulk density [g/cm³]	Swelling index [−]	Moisture content [%]	Gross calorific value [kJ/g]	Cyanide content [ppm]	Acidity content [g/100 g]
Agoè I	739	0.613	3.56	6.55		1.6	1.27
Agoè II	738	0.625	3.59	6.32		1.8	0.82
Agoè III	869	0.606	3.58	6.75	15.62	0.4	1.11
Agoè IV	741	0.667	4.33	7.10		0.9	0.73
Davie I	782	0.699	4.13	6.15		0.7	0.60
Davie II	664	0.690	4.07	6.90	15.50	2.4	1.33
Gapé‐Batekpo	694	0.625	3.50	4.30		0.4	0.57
Gapé‐Nyassivé	666	0.633	3.54	4.63	15.81	3.5	0.66
Tabligbo I	750	0.633	3.67	8.28	15.45	1.9	1.04
Tabligbo II	761	0.645	3.42	8.15		0.5	0.99
Tabligbo III	685	0.667	3.33	9.00		2.7	1.62
Vogan I	576	0.645	3.81	7.18		2.0	0.97
Vogan II	960	0.541	3.46	7.62	15.74	2.0	1.10
Vogan VI	557	0.606	3.21	9.40		0.4	1.05

The different samples comprise medians between 520 *μ*m and 943 *μ*m and weighted means from 557 *μ*m to 960 *μ*m. All samples are unclassified according to the Codex Alimentarius (Food and Agriculture Organization of the United Nations and World Health Organization 1989) and contain different fractions of grain sizes, which is typical for a Togolese market. Furthermore, the influence of the sieving time was controlled by using the analysis of variances and could not be proved to be significant (*P* ≥ 0.9). Moreover, the weighing accuracy can have a high influence on the result. The found bulk densities varied among 0.541 and 0.699 g/cm³. Again the weighing accuracy has an impact on these values. Finally, the swelling indices lay between 3.21 and 4.33.

The measured samples showed moisture contents between 4.30 and 9.40% with standard deviations from ±0.20 to ±0.74%. These measurements are dependent on the weighing accuracy, the sufficient drying time, and also the storage of the sample. Remarkable is the fact, that the moisture content increased after 5 months of storage, but there was no information about the original age of the samples available, which would be necessary to draw a conclusion out of this observation.

The gross calorific values resulted in energy contents between 15.45 and 15.81 kJ/g with standard deviations from ±0.19 to ±0.96 kJ/g. For the measurement, the weighing accuracy is important. Moreover, the definition of the calorimeter constant and the gross calorific value of the light fuel oil can have an influence on the result. Finally, there is also a device error within the temperature measurement.

The individual cyanide values were between 0 and 4.8 ppm. Noteworthy is the fact that the comparison with the color chart shows only a rough evaluation of the criticality. On the other hand, also the photometric determination delivered results with high variances. Presumably the method can be improved by using high‐quality laboratory equipment instead of the kit material as far as available. Especially, a high weighing accuracy and secured tightly closing vessels are indispensable.

The found acidity contents varied within a field from 0.57 to 1.33 g/100 g with appearing standard deviations between ±0.01 and ±0.08 g/100 g. Errors within this measurement can occur due to weighing insufficiencies, the measurement of the titration volume, or the determination of the color change. Moreover, the method expresses the total titrable acidity in percentage of lactic acid because this is the predominant acid in gari. Still it has to be kept in mind that other possible acids such as acetic acid are not taken into account.

**Figure 4:**
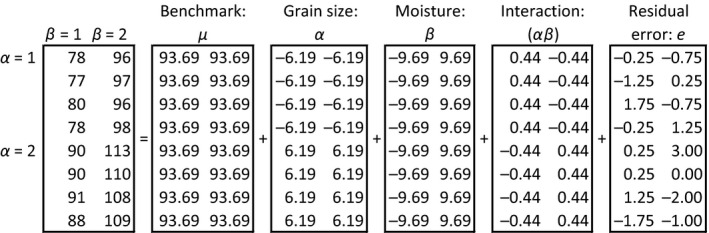
Decomposition of the dataset for the correlation tests.

After the moisture correction of the measured values out of the correlations tests, these values are compared to each other in order to find significant differences between the varied levels of the two treatment factors. Therefore, the found dataset first of all needs to be decomposed as shown in Figure 4. The factor grain size is named *α* and varied at level 1 (fine) and level 2 (coarse). The factor moisture content is named *β* and differed on level 1 (dry) and level 2 (humid). All acidity contents are multiplied with 100 for a better demonstration.

**Figure 5:**
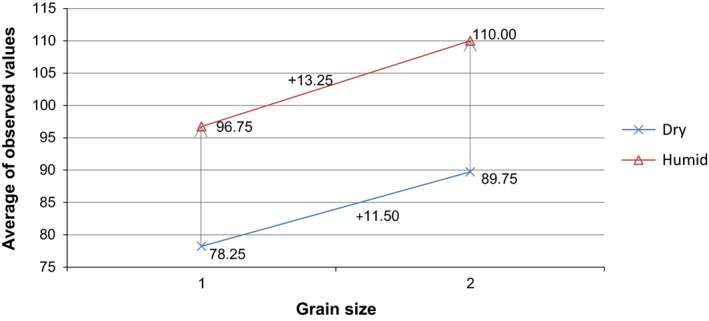
Interaction graph of the grouped averages for fine and coarse grains and dry and humid samples.

The analysis of variances for the possible correlations showed detectable effects for the factors grain size and moisture content, whereas the influence of an interaction effect was too small for a determination (*P* ≥ 99.9%). Comparisons of all factors showed that the parameter grain size and the parameter moisture content both have a significant impact on the acidity content. Evidently, the influence of the moisture content is bigger than that of the grain size. Moreover, the impact of the moisture content bigger for coarse gari than for fine gari. Finally, the influence of the grain size on dry gari is the smallest impact of all. The interaction graph in Figure 5 shows these interrelations through the grouped averages.

The correlation between the two parameters and its impact on the acidity content could not be proved, although the difference between dry, fine gari and humid, coarse gari is very high. As the analysis of variances showed, an extended model including more trials would be necessary to draw a clear conclusion about the interaction between coarse grains and high moisture content and their influence on the acidity content of gari.

## Conclusion

The described methods can be established as standard methods for the determination of the quality parameters of gari because they have low requirements to the available laboratory equipment. For some of them the accuracy can be improved by a higher quality of the measuring tools. Compared to gari from other countries in West Africa, the samples from the south of Togo showed all parameters within a common range, which implies the usability of the methods. The methods can be used to prove interactions between the individual parameters, therefore detailed correlation tests with parameters varied on different levels and several replications of each measurement are necessary. The cognition of these relations is also essential for conclusions out of single measurements. For example, the acidity content should never be computed without a moisture correction. In order to compare different samples, detailed knowledge of all parameters and their correlations among each other is indispensable to draw sustainable conclusions. Moreover, it is obligatory to have sufficient knowledge about the origin of the used cassava roots, the growing and harvesting conditions, the production process of gari, and storage conditions and storage period to understand how to improve the quality of gari. As the quality of gari does mainly depend on the processing methods, the difference of several harvest years can be regarded as negligible for the reliability of the methods.

On a long‐term view, these methods can help to improve the processing methods of cassava into gari and the quality of the final product. For example, the final product not only can be adapted to the individual African markets but also international markets. It could be produced in less time or work intensive and be safer according to its harmfulness caused by its cyanide content. These improvements can help to face the problems of malnutrition and poor living conditions of many people in the rural regions in West Africa.

## Conflict of Interest

None declared.
